# Time to surgery and its impact on length of stay and mortality for neck of femur fractures at a major trauma centre

**DOI:** 10.1007/s00068-026-03313-2

**Published:** 2026-08-24

**Authors:** Linda Yuhan Tang, Sharon Yuen Shan Ho, Andrew Kailin Zhou, Rahul Geetala, Matija Krkovic

**Affiliations:** 1 School of Clinical Medicine, University of Cambridge, Cambridge, CB2 0SP UK; 2 Department of Trauma and Orthopaedics, Addenbrookes Major Trauma Unit, Cambridge University Hospitals, Cambridge, UK; 3 University of Warwick, Warwick, UK; 4 Vascular Surgery Department, University Hospital Coventry and Warwickshire, Coventry, UK; 5 Manchester Foundation Trust, Manchester, UK

**Keywords:** Trauma, Fractures, NOF, Hip, Lower limb, Mortality, Time to surgery, Length of stay

## Abstract

**Purpose:**

Neck of femur (NOF) fractures are a major public health concern. Timely surgical intervention (current guidelines is within 36 h) is associated with improved survival, shorter hospital stays, and better functional outcomes. However, surgical delays remain common in hospitals, especially in major trauma centres, and their impact on length of stay (LOS) and mortality is not well defined across different NOF fracture subtypes.

**Methods:**

We conducted a retrospective cohort study of 4415 patients with NOF fractures, classified into intracapsular (*n* = 2507) and extracapsular (*n* = 1908). The primary outcome data gathered were LOS (from surgery to discharge) and in-hospital mortality. Regression analyses were performed to assess associations between LOS, and mortality, adjusting for age, sex, Charlson Comorbidity Index (CCI), American Society of Anesthesiologists (ASA) score, intra-operative blood transfusion, and surgical methods.

**Results:**

For intracapsular fractures, delays in time to surgery were significantly associated with increased LOS in patients expected to have short to median lengths of stay, after adjusting for other variables (*p* < 0.05). Delayed surgery was associated with increased mortality for both intracapsular and extracapsular fractures (*p* < 0.0001). Survival curves showed significant differences for the surgical delayed group. After controlling for time to surgery, ASA, CCI, intra-operative blood transfusion and surgical methods, patients ≥ 70 experienced longer LOS (*p* < 0.0001).

**Conclusion:**

Early surgery is associated with shorter LOS for intracapsular NOF fractures. Delayed surgery (> 36 h) significantly increases mortality risk for both intracapsular and extracapsular fractures. Patients ≥ 70 years face longer hospital stays and higher mortality, suggesting a need for targeted perioperative strategies. Our findings support prioritising high-risk patients for expedited surgery. Future work aims to develop a risk stratification tool to optimise surgical prioritisation.

**Supplementary Information:**

The online version contains supplementary material available at https://doi.org/10.1007/s00068-026-03313-2.

## Introduction

Neck of femur (NOF) fractures are a significant public health concern, particularly in older adults, due to their association with high morbidity, mortality, and healthcare costs. Globally, the incidence of NOF fractures is expected to rise with aging populations, further straining healthcare systems [1, 2]. Central to optimising outcomes is timely surgical intervention, which has been unequivocally shown to reduce mortality, enhance functional recovery, and minimise the length of hospital stay [3, 4].

Within healthcare systems such as the NHS in England, the adoption of “Best Practice Tariffs” (BPTs) underscores the commitment to delivering evidence-based, high-quality care for NOF fracture patients [5]. These tariffs incentivise key components of optimal management, most notably the achievement of surgery within 36 h of admission [5]. Early surgical intervention is not only associated with improved survival rates but also mitigates the risks of complications, thereby enhancing both clinical outcomes and healthcare efficiency [6, 7].

Despite well-established principles, delays to surgery remain a pervasive challenge in trauma care, often arising from multifactorial causes, including the need for medical optimisation, resource limitations, and the prioritisation of other surgical services in tertiary major trauma centres [8]. While the relationship between surgical timing and overall outcomes is well-recognised, the specific impact of surgical delay on hospital length of stay (LOS) warrants further investigation, particularly in tertiary trauma centres managing complex and resource-intensive caseloads.

This retrospective cohort study aims to address this critical gap by evaluating the association between time to surgery and LOS for patients with NOF fractures treated at a major trauma centre. By elucidating the impact of time to surgery from admission on LOS, this study aims to inform clinical decision-making, enhance resource allocation, and reinforce adherence to evidence-based guidelines, ultimately contributing to improved outcomes for this vulnerable patient population.

## Methods

### Study design

This was a retrospective cohort study conducted at a major trauma centre in the UK. Data were extracted from the hospital’s electronic patient record system (EPIC™) for all patients diagnosed with a hip fracture and underwent surgery between 1st of October 2014 to 31st May 2025 as part of an audit. The audit (PRN12941) was approved by the local Patient Outcomes Team (Audit Team), Clinical Project ID6941.

### Patient selection

Patients were included if they were diagnosed with a NOF fracture and underwent surgical fixation or arthroplasty. Exclusion criteria included patients younger than 18 years of age, those who died prior to surgery, those who underwent more than one surgical procedure during admission, those who were managed nonoperatively, those who received fixation for intracapsular fractures or arthroplasty for extracapsular fractures and those who had incomplete data on time to surgery, length of hospital stay, or their American Society of Anaesthesiologists (ASA) status.

### Definition and coding

Fractures were classified based on radiographic assessment and clinical coding as intracapsular or extracapsular NOF fractures. The surgical procedures performed included mainly hemiarthroplasty or total hip arthroplasty for intracapsular fractures, and intramedullary nailing or dynamic hip screw fixation for extracapsular fractures. All patients received standardised perioperative care. Postoperative rehabilitation followed an early mobilisation protocol, with weight-bearing as tolerated. All patients received standardised orthogeriatric care during their hospital stay.

### Data collection and outcomes

The primary outcome data gathered for this audit were time to surgery and length of hospital stay. Time to surgery was defined as the interval between hospital admission and the recorded start time of surgery in the operating theatre. LOS was defined as the time from surgery in the operating theatre to discharge. Secondary outcome data collected included postoperative complications and in-hospital mortality. Demographic and clinical data collected included age, sex, ethnicity, smoking, Charlson comorbidity index (CCI), ASA status, intra-operative transfusion volume, and surgical method.

### Statistical analysis

Descriptive statistics were used to summarise baseline characteristics. Continuous variables were assessed for normality and compared using Kruskal or Mann-Whitney U tests. To address missing values in ASA status, multiple imputation by chained equations (MICE) was performed using the mice package (version 3.19.0) in R (version 4.6.1), generating m = 20 imputed datasets. ASA status was imputed using ordered logistic regression, reflecting its ordinal nature. To assess the association between time to surgery and hospital length of stay (LOS), an quantile regression model was fitted separately on each of the 20 imputed datasets using the rms package (version 8.1.1) and quantreg package (version 6.1) in R, adjusting for age on admission, sex, ASA status, Charlson Comorbidity Index (CCI), intra-operative transfusion volume, and surgical types, while excluding patients who died during admission. Estimates were pooled across the 20 imputed datasets using Rubin’s rules (via fit.mult.impute) to obtain combined regression coefficients, standard errors, and 95% confidence intervals that account for uncertainty due to missing data. Ethnicity, smoking history and alcohol status is removed from the model due to insufficient data. The quantile regression model was specified as the following: time_of_stay ~ time_to_surgery + age_on_admission + sex + ASA_status + charlson + transfusion_volume + surgical_type for those without non-linear effects. Restricted cubic spline is used to model the non-linear effect when present. The plots showing linear and non-linear effects of the relevant variables are shown in Supplementary Figure 1. Confidence intervals of the model are calculated with simultaneous confidence intervals to correct for multiple comparisons. Within the intracapsular and extracapsular subgroups, we also explored whether there is any statistical significance between those who survived and those who died, using Mann-Whitney U test for continuous variables and Chi-Square test for categorical variables. Survival analysis is further conducted to plot Kaplan-Meier curves and adopting log-rank test and Cox proportional hazards model to adjust for confounding factors and calculating the hazard ratio and its 95% CI of surgical timing for survival.

## Results

### Patients and injury characteristics

14,153 patients with closed lower limb fractures were found from the hospital’s electronic health record. 852 patients were removed due to being below 18 years old and 4,547 patients were removed as they had more than 1 operation. After excluding all non-NOF fractures, we were left with 5,133 patients. Following the exclusion of cases with missing data, including only patients where there is ‘total hip replacement’ and ‘hemiarthroplasty’ in intracapsular fracture, and removing patients who received fixation for intracapsular fractures or arthroplasty for extracapsular fractures, 1,908 patients remained in the extracapsular group and 2,507 patients in the intracapsular group. The demographic data breakdown is shown in Table 1. Supplementary Table 1 shows comparisons of general patient characteristics between the intra- and extra-capsular groups.

**Table 1:** Demographic features and injury characteristics across the patients included

	Total (*n* = 4415)	
	Intracapsular fracture (*n* = 2,507)	Extracapsular fracture (*n* = 1,908)
**Age**		
- Range	43–102	18–105
- Mean ± s.d.	82.3 ± 9.3	81.8 ± 12.3
**Gender**		
- Female	1732 (69.1%)	1323 (69.3%)
- Male	775 (30.9%)	585 (30.7%)
**Ethnicity**		
- White British	2083 (83.1%)	1535 (80.5%)
- Other	81 (3.23%)	82 (4.30%)
- Unrecorded	333 (13.3%)	291 (15.3%)
**Smoking**		
- Yes	350 (14.0%)	276 (14.5%)
- No	389 (15.5%)	235 (12.3%)
- Not documented	1768 (70.5%)	1397 (73.2%)
**Alcohol use**		
- Yes	314 (12.5%)	203 (10.6%)
- No	280 (11.2%)	226 (11.8%)
- Unrecorded	1913 (76.3%)	1479 (77.5%)
**Comorbidities**		
- 0	668 (26.6%)	500 (26.2%)
- 1	749 (29.9%)	578 (30.3%)
- 2	605 (24.1%)	424 (22.2%)
- 3 or more	485 (19.3%)	406 (21.3%)
**ASA**		
- 1	72 (2.87%)	59 (3.09%)
- 2	646 (25.8%)	428 (22.4%)
- 3	1389 (55.4%)	1098 (57.5%)
- 4	340 (13.6%)	272 (14.3%)
- 5	6 (0.24%)	3 (0.16%)
**Surgical Types**		
- Total hip replacement (THR)	285 (11.4%)	-
- Hemiarthroplasty	2222 (88.6%)	-
- Dynamic hip screw	-	1570 (82.3%)
- Nail	-	338 (17.7%)
**Admission death**		
- Total	101 (3.9%)	110 (5.9%)
- Female	52 (2.1% of total, 51.5% of all deaths)	64 (3.4% of total, 58.1% of all deaths)
- Male	49 (2.0% of total, 48.5% of all deaths)	46 (2.4% of total, 41.8% of all deaths)

### LOS analysis

#### Intracapsular NOF fractures

The result is summarised in Table 2. The quantile R^2^ is 0.0783 (tau = 0.25), 0.0961 (tau = 0.50), and 0.0982 (tau = 0.75) with 2507 observations. The overall contribution of the predictors to the model was significant for all quantiles (*p* < 0.0001). Individually, age on admission, ASA status and the surgical type of total hip replacement with reference to hemiarthroplasty are significant across all quantiles. Of note, time to surgery is statistically significant in the 0.25 (*p* = 0.005) and 0.5 quantile (*p* = 0.021) but not the 0.75 quantile (*p* = 0.310), suggesting that for patients with shorter stays, time to surgery matters to the time they stay in hospital. However, for patients that are staying in hospital for a long time, time to surgery does not have a significant effect. Another note is that the number of CCIs are not significant at 0.25 quantile (*p* = 0.09) but significant at 0.5 (*p* < 0.001) and 0.75 (*p* < 0.001) quantile, suggesting that when the time of stay is short, the number of CCI does not affect the length of stay. This suggests that after adjusting for age, sex, ASA status, comorbidities, intra-operative blood transfusion, and surgical types, surgical timing was an independent predictor of LOS in this cohort. The estimated median impact of 1-hour increase in time to surgery will lead to a 6.06-hour increase in LOS (Table 2).

#### Extracapsular NOF fractures

The result is summarised in Table 2. The quantile R^2^ is 0.0740 (tau = 0.25), 0.0724 (tau = 0.50), and 0.0703 (tau = 0.75) with 1908 observations. The overall contribution of the predictors to the model was significant (*p* < 0.001). Individually, age on admission, ASA status and number of CCIs are statistically significant across all quantiles. Of note, time to surgery is only statistically significant at the 0.5 quantile (*p* = 0.03), suggesting that time to surgery has significant effect on the length of stay for patients who have a long length of stay. Intra-operative transfusion volume also has a statistically significant difference at the 0.25 quantile, suggesting transfusion volume could increase the length of stay of patients in shorter length of stays. The estimated median impact of 1-hour increase in time to surgery will lead to a 6.52-hour increase in LOS (Table 2).

**Table 2:** Multivariable quantile regression model for intracapsular and extracapsular NOF fractures and the effects on LOS.

Predictor	Coef (tau = 0.25)	*P*-value (tau = 0.25)	Coef (tau = 0.50)	*P*-value (tau = 0.5)	Coef (tau = 0.75)	*P*-value (tau = 0.75)	Effect (95% CI)
**Intracapsular**							
Intercept	-33.2	0.103	-20.5	0.574	62.0	0.283	-
Time to surgery	0.26	0.005	0.27	0.021	0.33	0.310	6.06 (0.901; 11.2)
Age on admission	2.02	< 0.001	2.37	< 0.001	1.98	0.006	28.4 (18.2; 38.7)
ASA status	13.74	< 0.001	28.7	< 0.001	48.4	< 0.001	28.7 (17.2; 40.1)
CCI	5.17	0.092	15.7	< 0.001	37.1	< 0.001	31.4 (16.1; 46.8)
Intra-operative transfusion volume	0.02	0.674	-0.02	0.836	0.09	0.775	-23.3 (-244; 197)
Sex (Female)	3.03	0.503	-4.90	0.529	-17.5	0.202	4.90 (-10.4; 20.2)
Surgical type (Total hip replacement)	56.31	< 0.001	-91.2	< 0.001	-137	< 0.001	-91.2 (-109; -73.4)
**Extracapsular**							
Intercept	-110	< 0.001	-108	< 0.001	-83.9	0.058	-
Time to surgery	0.13	0.372	0.27	0.038	0.26	0.055	5.52 (0.314; 10.7)
Age on admission	2.64	< 0.001	2.21	< 0.001	2.61	< 0.001	28.7 (20.4; 36.9)
ASA status	38.1	< 0.001	76.8	< 0.001	104	< 0.001	54.3 (41.7; 66.9)
ASA status (non-linear)	39.3	< 0.001	-90.3	< 0.001	-95.7	0.006	-
CCI	12.9	< 0.001	23.6	< 0.001	41.5	< 0.001	47.3 (29.9; 64.7)
Intra-operative transfusion volume	0.09	0.009	0.03	0.454	0.01	0.894	31.7 (-51.2; 115)
Surgical type (intramedullary nail)	-16.1	0.1	0.67	0.942	-7.52	0.739	-0.671 (-18.7; 17.4)
Sex (Female)	3.21	0.671	4.17	0.659	-7.69	0.701	-4.17 (-22.7; 14.3)

Coef represents the coefficient of the parameter in the model, tau refers to quantiles. Effect refers to the effect size of the parameter affected by the length of stay i.e. how much does increase in 1 unit of the length of stay increases the value of such parameter. P-value is taken as statistically significant for *p* < 0.05. The reference categories for surgery type were hemiarthroplasty for the intracapsular group, and dynamic hip screw for the extracapsular group

### Comparing between Dead and Alive – inpatient mortality

We looked at the general patient characteristics across the intracapsular and extracapsular groups, and the significant difference between those who died and survived (Supplementary Table 2a). For both groups, the patient population that died has a higher mean age, higher mean ASA, higher CCI, longer time to surgery and more blood transfusion. Time to surgery is a significant factor determining whether a patient would die in an admission (*p* < 0.0001). Comparing the number of patients for each surgical type in patients who have survived and died, a Chi-Square test shows that there are significant differences between the 2 groups for intracapsular (χ² = 8.26, *p* = 0.00405) but not in extracapsular (χ² = 0.00, *p* = 1.0) (Supplementary Table 2b).

When the number of patients who died is stratified by the time to surgery (≤ 36 h and > 36 h), a Chi-Square test is completed which shows that there is significant difference in the number of deaths between time to surgery groups for intracapsular fractures (χ² = 22.5, p = < 0.0001) and extracapsular fractures (χ² = 5.82, *p* = 0.0158) (Supplementary Table 3).

**Fig. 1:**
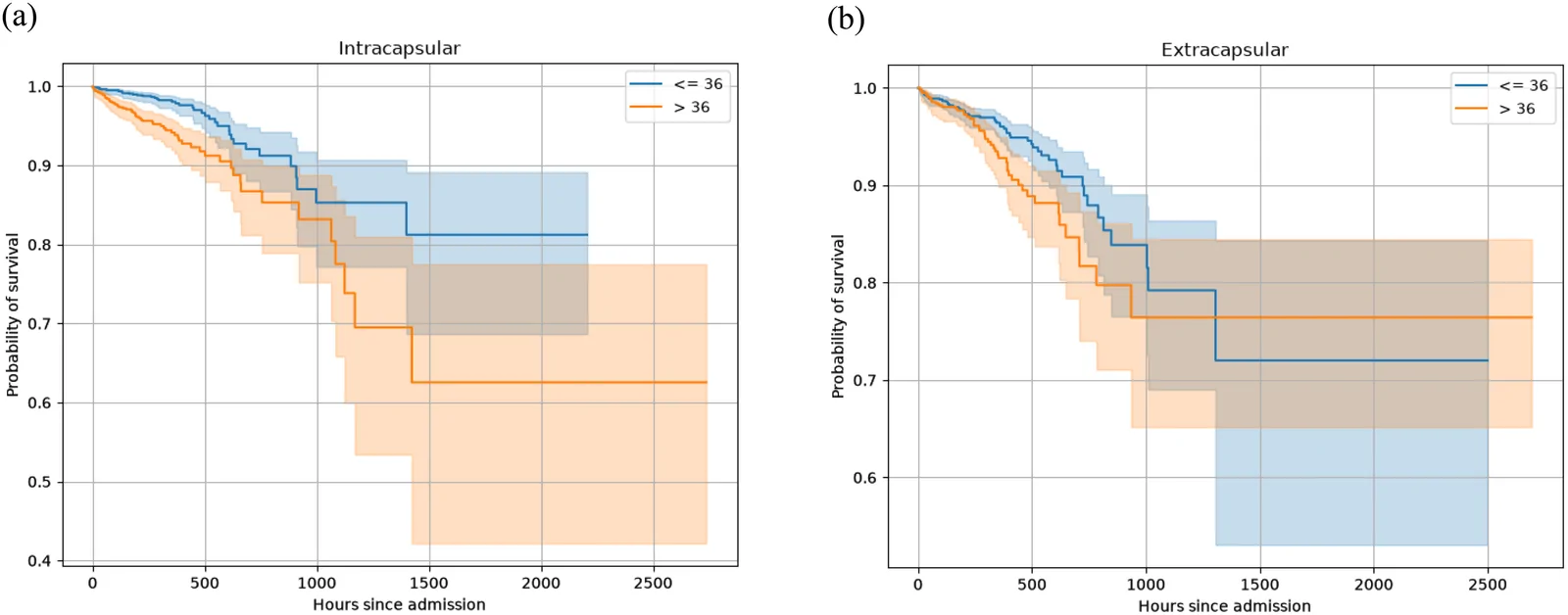
Kaplan-Meier survival curve of (**a**) intracapsular and (**b**) extracapsular fractures. Both graphs show that the survival of patients with a delay of over 36 h is lower, but the effect is more significant in the intracapsular fracture group

Kaplan-Meier survival curves for intracapsular (Fig. 1a) and extracapsular (Fig. 1b) fractures were generated to compare in-hospital survival between patients undergoing surgery within 36 h and those delayed beyond 36 h. Patients with delays > 36 h showed lower survival probabilities over time. Survival differed significantly between the surgery delay group for both intracapsular (log-rank test χ² = 20.6, *p* < 0.005) and extracapsular fractures (log-rank test χ² = 5.03, *p* = 0.02). In the multivariable Cox proportional hazards model adjusting for age, ASA status, Charlson comorbidity index, intra-operative transfusion volume, and surgical type (Table 3), surgical delay > 36 h was associated with increased in-hospital mortality for intracapsular fractures (HR 2.03, 95% CI 1.36–3.05, *p* = 0.0006) but not in extracapsular fractures (HR 1.43, 95% CI 0.978–2.10, *p* = 0.0646).

**Table 3:** Hazard ratio of Cox Regression Model for both intracapsular and extracapsular fractures for the ≤ 36 h and > 36 h surgical delay groups

Predictor	HR (95% CI)	*p*-value
**Intracapsular**		
Time to surgery	2.03 (1.36; 3.05)	0.0006
Age on admission	1.03 (1.00; 1.06)	0.0241
ASA status	1.84 (1.28; 2.64)	0.0010
CCI	1.50 (1.31; 1.72)	0.0000
Intra-operative transfusion volume	1.00 (0.998; 1.00)	0.868
Surgical type (Total hip replacement)	1.56 (0.361; 6.76)	0.550
**Extracapsular**		
Time to surgery	1.43 (0.978; 2.10)	0.0646
Age on admission	1.03 (1.01; 1.06)	0.0122
ASA status	2.10 (1.51; 2.94)	0.0000
CCI	1.30 (1.14; 1.49)	0.0001
Intra-operative transfusion volume	1.00 (1.00; 1.00)	0.241
Surgical type (intramedullary nail)	0.996 (0.605; 1.64)	0.987

Analysis performed for the > 36 h subgroup in reference to the ≤ 36 h subgroup. P-value is taken as statistically significant for *p* < 0.05

## Stratification by length to surgery (≤ 36 h and > 36 h)

A Mann-Whitney U test was done to investigate the general patient characteristics across the intracapsular and extracapsular groups, and if they are significantly different between those who received surgery below 36 h and those who received surgery after 36 h (Supplementary Table 4a). For the intracapsular fractures group, ASA status (*p* = 0.0003), CCI (*p* = 0.009), and age on admission (*p* = 0.05) is significantly different between the two time to surgery groups. For extracapsular fractures, there is a significant difference between both ASA status (*p* < 0.0001) and CCI (*p* < 0.0001). Comparing the number of patients for each surgical type when stratified by time to surgery, a Chi Square test shows that there is significant difference for the intracapsular fractures group (χ² = 9.54, *p* = 0.002) and the extracapsular fractures group (χ² = 5.27, *p* = 0.022) (Supplementary Table 4b).

### Surgical timing stratification models – surgical timing and impact on outcomes

#### Patients with time to surgery under 36 h with intracapsular NOF fractures

For this model, the quantile R^2^ is 0.0809 (tau = 0.25), 0.0907 (tau = 0.0907), and 0.0909 (tau = 0.0909) with 1610 observations. The overall contribution of the predictors to the model was significant (*p* < 0.001). Individually, age on admission, ASA status, and surgical type total hip replacement with reference to hemiarthroplasty is statistically significant across all quantiles. However, the number of CCIs is not statistically significant at the 0.25 quantile suggesting that the number of CCI doesn’t play a role in length of stay in short length of stays. In this group, both CCI, ASA status and age on admission has a large effect on increasing the length of stay, whilst total hip arthroplasty markedly reduces the length of stay compared to hemiarthroplasty (Table 4).

#### Patients with time to surgery over 36 h with intracapsular NOF fractures

For this model, the quantile R^2^ is 0.0677 (tau = 0.25), 0.100 (tau = 0.5) and 0.105 (tau = 0.75) with 897 observations. The overall contribution of the predictors to the model was significant (*p* < 0.001) across all quantiles. Surgical type of total hip replacement compared to hemiarthroplasty and ASA status is statistically significant across all quantiles. Age on admission is only statistically significant in 0.25 and 0.50 quantile suggesting that age is playing a significant role in affecting the length of stay in shorter length of stay. Number of CCI is not statistically significant in 0.25 quantile, suggesting that number of CCIs are not playing a significant role in affecting the length of stay in those patients with a shorter length of stay. Intra-operative transfusion volume is statistically significant in longer length of stays suggesting that for patients with a long length of stay, having a transfusion is associated with reduced time of stay (Table 4).

#### Patients with time to surgery under 36 h with extracapsular fractures

For this model, the quantile R^2^ is 0.0821 (tau = 0.25), 0.0734 (tau = 0.5), and 0.0759 (tau = 0.75) with 1284 observations. The overall contribution of the predictors to the model was significant across all quantiles (*p* < 0.001) across all quantiles. Age on admission and number of CCIs are statistically significant across all quantiles. ASA status is statistically significant at 0.75 quantile but not 0.25 and 0.5 quantile, suggesting that ASA status plays a role in affecting length of stay in higher length of stay. Surgical type of nail with reference to dynamic hip screw is statistically significant only in 0.25 quantile suggesting that in patients with a short length of stay, surgical types plays a role in the length of stay (Table 4).

#### Patients with time to surgery above 36 h with extracapsular fractures

For this model, the quantile R^2^ is 0.0520 (tau = 0.25), 0.0448 (tau = 0.50), and 0.0419 (tau = 0.75) with 624 observations. The overall contribution of the predictors to the model was significant across all quantiles (*p* < 0.001). Age on admission and ASA status is only statistically significant on quantile 0.25 and 0.5, suggesting that it plays a significant role in people with shorter length of stay. In contrast, CCIs are only statistically significant in the 0.5 and 0.75 quantile, suggesting that it has a significant effect on people with a longer length of stay (Table 4).

**Table 4:** Multivariable quantile regression model for intracapsular and extracapsular NOF fractures and the effects on LOS stratified by time to surgery

Predictor	Coef (tau = 0.25)	*P*-value (tau = 0.25)	Coef (tau = 0.50)	*P*-value (tau = 0.5)	Coef (tau = 0.75)	*P*-value (tau = 0.75)	Effect (95% CI)
**Intracapsular NOF fractures with surgery under 36 h**							
Intercept	-58.6	0.047	-79.1	0.069	52.4	0.488	
Age on admission	2.48	< 0.001	3.17	< 0.001	2.32	0.013	38.0 (25.8; 50.2)
ASA status	11.8	0.033	26.7	< 0.001	38.6	0.005	26.7 (12.7; 40.8)
CCI	7.71	0.068	19.6	< 0.001	46.2	< 0.001	39.2 (19.9; 58.5)
Intra-operative transfusion volume	0.02	0.751	0.07	0.434	0.03	0.822	42.7 (-64.4; 150)
Sex (Female)	1.83	0.806	-8.37	0.388	-12.8	0.504	8.37 (-10.6; 27.4)
Surgical (Total hip replacement)	-49.4	< 0.001	-75.8	< 0.001	-127	< 0.001	-75.8 (-96.8; -54.8)
**Intracapsular NOF fractures with surgery over 36 h**							
Intercept	38.5	0.289	57.3	0.349	161	0.111	
Age on admission	1.14	0.012	1.44	0.05	0.93	0.453	18.7 (-<0.01; 37.4)
ASA status	19.81	0.007	38.5	< 0.001	54.4	< 0.001	38.5 (18.6; 58.4)
CCI	1.27	0.801	16.3	0.014	32.6	0.007	32.6 (6.72; 58.5)
Intra-operative transfusion volume	-0.03	0.899	-0.06	0.850	0.37	0.012	-61.8 (-701; 578)
Sex (Female)	-1.21	0.887	-15.9	0.233	-16.9	0.435	15.9 (-10.2; 41.9)
Surgical (Total hip replacement)	-63.4	< 0.001	= 98.7	< 0.001	-154	< 0.001	-98.7 (-129; -68.9)
**Extracapsular NOF fractures with surgery under 36 h**							
Intercept	-113	< 0.001	-139	< 0.001	-94.3	0.204	-
Age on admission	2.81	< 0.001	2.73	< 0.001	2.42	0.007	36.2 (25.7; 46.7)
ASA status	46.2	< 0.001	99.3	< 0.001	136	0.004	23.1 (5.82; 40.4)
ASA status (non-linear)	-53.5	0.002	130	< 0.001	-117	0.146	-
CCI	14.2	0.001	33.0	< 0.001	45.3	< 0.001	42.4 (10.8; 74.0)
Intra-operative transfusion volume	0.11	< 0.001	0.05	0.228	0.02	0.806	65.0 (-21.4; 151)
Sex (Female)	-0.78	0.924	-7.48	0.528	2.61	0.266	5.67 (-18.4; 29.7)
Surgical type (intramedullary nail)	-24.5	0.003	-15.0	0.24	-33.6	0.918	10.1 (-13.8; 33.9)
**Extracapsular NOF fractures with surgery over 36 h**							
Intercept	-102	0.002	-141	< 0.001	-82.7	0.671	-
Age on admission	1.85	0.001	1.54	0.014	2.04	0.179	21.5 (4.33; 38.8)
ASA status	62.8	< 0.001	128	< 0.001	121	0.218	167 (77.8; 256)
ASA status (non-linear)	-51.3	0.059	-129	< 0.001	-93.6	0.479	-
CCI	10.5	0.088	22.1	0.001	42.4	0.003	27.6 (10.7; 44.5)
Intra-operative transfusion volume	-0.02	0.769	0.02	0.792	-0.01	0.92	23.9 (-154; 202)
Surgical type (intramedullary nail)	0.58	0.970	12.0	0.452	38.8	0.293	-12.0 (-43.3; 19.3)
Sex (Female)	5.38	0.707	10.0	0.495	-14.7	0.684	10.0 (-38.8; 18.8)

Coef represents the coefficient of the parameter in the model, tau refers to quantiles. Effect refers to the effect size of the parameter affected by the length of stay i.e. how much does increase in 1 unit of the length of stay increases the value of such parameter. P-value is taken as statistically significant for *p* < 0.05. The reference categories for surgery type were hemiarthroplasty for the intracapsular group, and dynamic hip screw for the extracapsular group

## Stratification by age

A Mann-Whitney U test was done to investigate the general patient characteristics across the intracapsular and extracapsular groups, and if they are significantly different between those who are under 70 and those who are 70 or above (Supplementary Table 5a). For both groups, ASA, CCI, and LOS were significantly different between the under 70 and the 70 or above age groups (*p* < 0.0001). The time to surgery and the intra-operative transfusion volume were not significantly different between the under 70 and 70 or above groups (intracapsular cohort: *p* = 0.1 and *p* = 0.8 respectively; extracapsular cohort: *p* = 0.2 and *p* = 0.1 respectively). Comparing the number of patients for each surgical type when stratified by age, a Chi-Square test shows that there is significant difference between under 70 and over 70 for intracapsular fractures (χ² = 454, p = < 0.0001) and extracapsular fractures (χ² = 13.0, *p* = 0.0003) (Supplementary Table 5b).

### Age stratification models - age of patients and impact on outcomes

#### Patients under 70 with intracapsular NOF fractures

For this model, the quantile R^2^ is 0.0879 (tau = 0.25), 0.148 (tau = 0.50) and 0.222 (tau = 0.75) with 242 observations. Surgical type of total hip replacement compared to hemiarthroplasty is statistically significant across all quantiles. Time to surgery is only statistically significant in 0.25 and 0.5 quantile, suggesting that time to surgery is playing a significant role when patients have a shorter stay in hospital. ASA status is only statistically significant at the 0.5 quantile (Table 5).

#### Patients 70 or above with intracapsular NOF fractures

For this model, the quantile R^2^ is 0.0525 (tau = 0.25), 0.0676 (tau = 0.50) and 0.0748 (tau = 0.0748) with 2265 observations. The overall contribution of the predictors to the model was significant across all quantiles (*p* < 0.001). ASA status and surgical type total hip replacement with reference to hemiarthroplasty is statistically significant across all quantiles while number of CCI is not statistically significant for the 0.25 quantile (*p* = 0.204), suggesting that the number of CCI plays a statistically significant role on increasing the length of stay only at longer length of stays (Table 5).

#### Patients under 70 with extracapsular NOF fractures

For this model, the quantile R^2^ is 0.122 (tau = 0.25), 0.160 (tau = 0.50), and 0.209 (tau = 0.75) with 245 observations. The overall contribution of the predictors to the model was significant (*p* < 0.001) across all quantiles. Number of CCIs is statistically significant across all quantiles. Time to surgery is only statistically significant at 0.5 quantile, suggesting that in this group the time to surgery is playing a larger role in people with median length of stay. ASA status is also statistically significant at 0.5 quantile (Table 5).

#### Patients 70 or above with extracapsular NOF fractures

For this model, the adjusted R^2^ is 0.0350 with 1663 observations. The overall contribution of the predictors to the model was significant (*p* < 0.001) across all quantiles. ASA status is statistically significant across all quantiles. Time to surgery is only statistically significant at 0.75 quantile (*p* < 0.001) suggesting that time to surgery is only significant for longer length of stay. Being female over male is also statistically significant at 0.5 quantile for having a higher length of stay (Table 5).

**Table 5:** Multivariable quantile regression model for intracapsular and extracapsular NOF fractures and the effects on LOS stratified by time to surgery

Predictor	Coef (tau = 0.25)	*P*-value (tau = 0.25)	Coef (tau = 0.50)	*P*-value (tau = 0.5)	Coef (tau = 0.75)	*P*-value (tau = 0.75)	Effect (95% CI)
**Intracapsular NOF fractures**,** patients under 70**							
Intercept	101	< 0.001	141	< 0.001	269	< 0.001	
Time to surgery	0.40	0.015	0.38	< 0.001	0.10	0.815	8.61 (4.60; 12.6)
ASA status	9.98	0.101	19.3	0.026	20.8	0.226	19.4 (2.42; 36.3)
CCI	5.76	0.436	16.2	0.128	23.9	0.357	16.2 (4.66; 37.1)
Intra-operative transfusion volume	0.02	0.900	-0.05	0.928	-0.09	0.897	-58.1 (8.20; 1200)
Sex (Female)	6.23	0.460	-1.99	0.867	-3.55	0.836	1.91 (1.39; 25.2)
Surgical (Total hip replacement)	-54.5	< 0.001	-83.5	< 0.001	-167	< 0.001	83.3 (0.467; 126)
**Intracapsular NOF fractures**,** patients 70 or above**							
Intercept	122	< 0.001	160	< 0.001	187	< 0.001	
Time to surgery	0.23	0.073	0.21	0.137	0.41	0.224	4.66 (-1.49; 10.8)
ASA status	20.0	< 0.001	36.7	< 0.001	63.2	< 0.001	36.7 (24.0; 49.3)
CCI	4.38	0.204	16.6	< 0.001	37.4	< 0.001	16.6 (8.53; 24.6)
Intra-operative transfusion volume	0.02	0.660	0.05	0.404	0.36	0.280	42.7 (-57.6; 143)
Sex (Female)	3.49	0.620	-5.80	0.507	-15.5	0.374	5.80 (-11.3; 22.9)
Surgical (Total hip replacement)	-75.4	< 0.001	-112	< 0.001	-162	< 0.001	-112 (-130; -94.6)
**Extracapsular NOF fractures**,** patients under 70**							
Intercept	65.4	0.002	37.9	0.290	69.4	0.155	
Time to surgery	0.14	0.784	0.51	0.004	0.49	0.051	12.1 (8.21; 16.1)
ASA status	14.9	0.210	54.1	0.025	67.5	0.051	40.1 (11.8; 68.4)
ASA status (non-linear)	11.4	0.682	-35.5	0.634	93.6	0.347	-
CCI	42.8	0.002	63.3	< 0.001	57.8	0.017	63.0 (37.0; 89.0)
Intra-operative transfusion volume	0.05	0.701	0.31	0.135	0.22	< 0.001	400 (-115; 915)
Sex (Female)	-4.37	0.578	-10.7	0.516	-11.1	0.742	-4.14 (-36.6; 28.4)
Surgical type (intramedullary nail)	-20.1	0.029	-25.4	0.203	-12.3	0.597	18.8 (-18.1; 55.7)
**Extracapsular NOF fractures**,** patients 70 or above**							
Intercept	-45.9	0.299	-96.9	0.054	-46.2	0.632	
Time to surgery	0.15	0.129	0.12	0.259	0.19	< 0.001	2.48 (-1.82; 6.78)
ASA status	116	< 0.001	171	< 0.001	205	< 0.001	141 (82.9; 200)
ASA status (non-linear)	-126	< 0.001	-203	< 0.001	-221	0.003	-
CCI	7.45	0.054	24.4	< 0.001	39.0	< 0.001	24.4 (15.7; 33.0)
Intra-operative transfusion volume	0.05	0.230	-0.01	0.773	0.00	0.979	-7.79 (-60.8; 45.2)
Sex (Female)	15.4	0.175	20.8	0.029	2.97	0.888	-20.8 (-39.5; -2.19)
Surgical type (intramedullary nail)	-8.88	0.393	17.9	0.082	7.28	0.769	-17.9 (-38.1; 2.27)

Coef represents the coefficient of the parameter in the model, tau refers to quantiles. Effect refers to the effect size of the parameter affected by the length of stay i.e. how much does increase in 1 unit of the length of stay increases the value of such parameter. P-value is taken as statistically significant for *p* < 0.05. The reference categories for surgery type were hemiarthroplasty for the intracapsular group, and dynamic hip screw for the extracapsular group

## Discussion

The findings of this study reinforce the importance of timely surgical intervention in patients with NOF fractures, particularly in relation to LOS, mortality and age. The results suggest that while adherence to the 36-hour BPT target is beneficial in reducing LOS and optimising patient outcomes [5], groups with higher ASA, CCIs, and advanced age, require further prioritisation for expedited surgical intervention. Although time to surgery was found to be a significant predictor of LOS, its effect size was relatively small compared to other significant factors, such as age, ASA status, and CCI. This suggests that while timely surgical intervention is beneficial, greater emphasis should be placed on optimising perioperative care for high-risk patients. The time to surgery cannot however be disregarded, as further findings suggest that there are significant differences in mortalities between patients who had surgery ≤ 36 h and > 36 h. Kaplan–Meier survival curves for intracapsular and extracapsular groups also showed significant survival differences relating to the time to surgery.

Further in-depth analysis with patients stratified by time to surgery (≤ 36 h and > 36 h) showed that for both intracapsular and extracapsular groups, ASA and CCI were significant factors that affect length of stay in patients with surgery ≤ 36 h and > 36 h. Compared to hemiarthroplasty, total hip replacement was found to be a significant factor in reducing length of stay in the intracapsular cohort, which seemed reasonable since hemiarthroplasty is usually performed on more frail patients. When patients were stratified by age (< 70 or ≥ 70), ASA was main predictors of length of stay in patients ≥ 70 years old in both intra- and extra-capsular fracture groups. Time to surgery was a significant predictor for intracapsular patients with short and median lengths of stay, and was a significant predictor for extracapsular patients with median or long lengths of stay, depending on the age of the patient. When exploring the factors that may affect patient mortality, it is found that for those who passed away, they had a higher age, had more comorbidities, and had a longer delay to surgery. This highlights the importance of implementing comprehensive, multidisciplinary preoperative optimisation and early rehabilitation strategies to improve overall outcomes.

Our findings align with existing joint guidelines from NICE and British Orthopaedic Association (BOA), and the National Hip Fracture Database (NHFD), all of which advocate for early surgery to optimise outcomes [5, 9]. Our analysis demonstrated that delayed surgery is associated with a significantly higher mortality risk in patients with NOF fractures. Additionally, we found that LOS was significantly prolonged in patients aged 70 or above, irrespective of fracture type.

The current literature supports our findings that prioritising high-risk patients for surgery within a shorter time frame could lead to improved outcomes and reduced LOS, and operations should not be delayed based on patient frailty or comorbidities alone [10]. The HIP ATTACK study however, did not find any significant reduction in adverse outcomes when patients had accelerated surgery, however, their median time to surgery was 6 h for the accelerated group, and 24 h for the standard care group, both less than the 36-hour cut-off that we were using for our study [11]. Our studies suggest that the current guidelines of a blanket 36-hour target for all NOF fractures may be insufficient for certain demographic groups, warranting further research in the future. Furthermore, the influence of patient demographics on surgical urgency is not explicitly addressed in current guidelines [5, 9]. Based on our findings, future revisions of guidelines should consider stratifying surgical urgency based on age, NOF fracture type, comorbidity burden, and ASA grade, rather than maintaining a uniform 36-hour target for all patients. Additionally, the implications of increased LOS within major trauma centre (MTC) settings, which often struggle with capacity and bed pressures, highlight the need for systemic changes to improve compliance with best practice targets [12].

### Implications for Best Practice Tariff guidelines

The NHS BPT incentivises surgery within 36 h to enhance patient outcomes and reduce hospital resource utilisation [5]. Our findings, consistent with prior literature [13–15], confirm the benefits of early surgical intervention in reducing LOS. However, our data suggest that for high-risk subgroups, delays in time to surgery may significantly exacerbate LOS and further elevate mortality risk. A tiered prioritisation framework could be considered, where high-risk patients are fast-tracked for operative management.

### Challenges for major trauma centres in meeting the 36-hour target

Despite strong evidence supporting early surgical intervention, MTCs face significant logistical challenges in consistently achieving the BPT target. MTCs frequently manage complex polytrauma cases, which require prioritisation over NOF fractures [12]. Resource limitations, including theatre availability and staffing constraints, further contribute to surgical delays [16].

The introduction of dedicated NOF operating lists within MTCs could facilitate improved compliance with guidelines, ensuring timely surgery while preserving capacity for critical emergencies. This approach could support equitable access to prompt surgical care, irrespective of the complexities associated with being treated in an MTC setting.

### Limitations

We acknowledge there are several limitations to our study. First, it is a retrospective cohort study of a single trauma centre. Second, data on pre-injury functional and cognitive status were unavailable, which may act as confounding factors. Third, surgical technique variability could not be controlled. And finally, the sample size for patients under 70 years old was relatively small, and for all patients, we only had access to their intra-operative rather than the post-operative transfusion volume data. However, we address these limitations by using a large patient cohort and multivariable regression models, aiming to reduce any confounding effects and searching for the predictors for LOS and mortality. Although our study was conducted at a single centre, our institution serves a demographically diverse population and the trust adheres to national hip fracture guidelines, improving external validity. To account for variations in surgical pathways and preoperative care, we stratified our analysis based on fracture type (intracapsular vs. extracapsular), ensuring that findings were relevant across different patient groups. Our study focuses on critical outcome predictors, specifically LOS and mortality [17]. Future multi-centre studies with standardised clinical procedures should be performed. Research should build on the findings in this study by incorporating additional patient-specific variables, such as frailty indices, post-operative transfusion volumes, post-discharge outcomes, and by developing a risk stratification scoring system to facilitate early surgical prioritisation of high-risk patients.

## Conclusion

This study highlights the critical role of timely surgical intervention in optimising clinical outcomes for patients with NOF fractures and underscores the need to refine current guidelines to better address the requirements of high-risk patient populations. The challenges faced by MTCs in achieving BPT targets suggest a need for structural changes, including the establishment of dedicated NOF operating lists or referral pathways to specialist NOF centres. Future research should explore the impact of these interventions on both patient outcomes and healthcare resource utilisation, ensuring that national guidelines continue to evolve towards supporting equitable and efficient hip fracture management.

## Supplementary Information

Below is the link to the electronic supplementary material.

Supplementary Material 1

## Data Availability

Data were extracted from the hospital’s electronic patient record system as part of an audit. The audit was approved by the local Patient Outcomes Team (Audit Team).

## References

[1] Abrahamsen B, van Staa T, Ariely R, Olson M, Cooper C. Excess mortality following hip fracture: a systematic epidemiological review. Osteoporos Int J Establ Result Coop Eur Found Osteoporos Natl Osteoporos Found USA. 2009;20(10):1633–50. 10.1007/s00198-009-0920-3 19421703

[2] Cauley JA. Public health impact of osteoporosis. J Gerontol Biol Sci Med Sci. 2013;68(10):1243–51. 10.1093/gerona/glt093 PMC3779634 23902935

[3] Moja L, Piatti A, Pecoraro V, Ricci C, Virgili G, Salanti G, et al. Timing matters in hip fracture surgery: patients operated within 48 hours have better outcomes. A meta-analysis and meta-regression of over 190,000 patients. PLoS ONE. 2012;7(10):e46175. 10.1371/journal.pone.0046175 PMC3463569 23056256

[4] Shiga T, Wajima Z, Ohe Y. Is operative delay associated with increased mortality of hip fracture patients? Systematic review, meta-analysis, and meta-regression. Can J Anaesth J Can Anesth. 2008;55(3):146–54. 10.1007/BF03016088 18310624

[5] Best practice tariff user guide.pdf [Internet]. [cited 2025 Mar 6]. Available from: https://www.nhfd.co.uk/20/hipfracturer.nsf/0/9b0c5ea2e986ff56802577af0046b1df/$file/best%20practice%20tariff%20user%20guide.pdf

[6] Holt G, Smith R, Duncan K, Finlayson DF, Gregori A. Early mortality after surgical fixation of hip fractures in the elderly: an analysis of data from the scottish hip fracture audit. J Bone Joint Surg Br. 2008;90(10):1357–63. 10.1302/0301-620X.90B10.21328 18827248

[7] Moran CG, Wenn RT, Sikand M, Taylor AM. Early mortality after hip fracture: is delay before surgery important? J Bone Joint Surg Am. 2005;87(3):483–9. 10.2106/JBJS.D.01796 15741611

[8] Pioli G, Giusti A, Barone A. Orthogeriatric care for the elderly with hip fractures: where are we? Aging Clin Exp Res. 2008;20(2):113–22. 10.1007/BF03324757 18431078

[9] Recommendations | Hip fracture: management | Guidance | NICE [Internet]. [cited 2025 Mar 6]. Available from: https://www.nice.org.uk/guidance/cg124/chapter/Recommendations

[10] Forssten MP, Mohammad Ismail A, Ioannidis I, Ribeiro MAF, Cao Y, Sarani B, et al. Prioritizing patients for hip fracture surgery: the role of frailty and cardiac risk. Front Surg [Internet]. 2024 Mar 8 [cited 2026 July 31];11. Available from: https://www.frontiersin.org/journals/surgery/articles/10.3389/fsurg.2024.1367457/full. 10.3389/fsurg.2024.1367457 PMC10957751 38525320

[11] Borges FK, Bhandari M, Guerra-Farfan E, Patel A, Sigamani A, Umer M, et al. Accelerated surgery versus standard care in hip fracture (HIP ATTACK): an international, randomised, controlled trial. Lancet. 2020;395(10225):698–708. 10.1016/S0140-6736(20)30058-1 32050090

[12] Kanakaris NK, Giannoudis PV. Trauma networks: present and future challenges. BMC Med. 2011;9(1):121. 10.1186/1741-7015-9-121 PMC3229440 22078223

[13] Fu MC, Boddapati V, Gausden EB, Samuel AM, Russell LA, Lane JM. Surgery for a fracture of the hip within 24 hours of admission is independently associated with reduced short-term post-operative complications. Bone Joint J. 2017 Sept 1;99-B(9):1216–22. 10.1302/0301-620X.99B9.BJJ-2017-0101.R1 28860403

[14] Yoo J, Lee JS, Kim S, Kim BS, Choi H, Song DY, et al. Length of hospital stay after hip fracture surgery and 1-year mortality. Osteoporos Int. 2019;30(1):145–53. 10.1007/s00198-018-4747-7 30361752

[15] Craigven SHS, Rehena S, Kenny TXK, Howe CY, Howe TS, Joyce KSB. Shorter acute hospital length of stay in hip fracture patients after surgery predicted by early surgery and mobilization. Arch Osteoporos. 2021;16(1):1–8. 10.1007/s11657-021-01027-z 34718871

[16] Wong J, Khu KJ, Kaderali Z, Bernstein M. Delays in the operating room: signs of an imperfect system. Can J Surg J Can Chir. 2010;53(3):189–95. PMC2878989 20507792

[17] Ek S, Meyer AC, Hedström M, Modig K. Hospital Length of Stay After Hip Fracture and It’s Association With 4-Month Mortality—Exploring the Role of Patient Characteristics. J Gerontol Biol Sci Med Sci. 2021;77(7):1472–7. 10.1093/gerona/glab302 PMC9255691 34622920

