# Correction: Time to surgery and its impact on length of stay and mortality for neck of femur fractures at a major trauma centre

**DOI:** 10.1007/s00068-026-03376-1

**Published:** 2026-10-26

**Authors:** Linda Yuhan Tang, Sharon Yuen Shan Ho, Andrew Kailin Zhou, Rahul Geetala, Matija Krkovic

**Affiliations:** 1 School of Clinical Medicine, University of Cambridge, Cambridge, CB2 0SP UK; 2 University of Warwick, Warwick, UK; 3 Vascular Surgery Department, University Hospital Coventry and Warwickshire, Coventry, UK; 4 Manchester Foundation Trust, Manchester, UK; 5 Department of Trauma and Orthopaedics, Addenbrookes Major Trauma Unit, Cambridge University Hospitals, Cambridge, UK

**European Journal of Trauma and Emergency Surgery (2026) 52:261**

**https://doi.org/10.1007/s00068-026-03313-2**

We recently discovered a transcription error in our published manuscript regarding Table 5, under the section “Intracapsular NOF fractures, patients 70 or above”. Below are the details of the error and the requested correction.

**The incorrect table (Table 5, on Page 8)**:

**Table:** 

Predictor	Coef (tau = 0.25)	P-value (tau = 0.25)	Coef (tau = 0.50)	P-value (tau = 0.5)	Coef (tau = 0.75)	P-value (tau = 0.75)	Effect (95% CI)
**Intracapsular NOF fractures, patients under 70**							
Intercept	101	<0.001	141	<0.001	269	<0.001	
Time to surgery	0.40	0.015	0.38	<0.001	0.10	0.815	8.61 (4.60; 12.6)
ASA status	9.98	0.101	19.3	0.026	20.8	0.226	19.4 (2.42; 36.3)
CCI	5.76	0.436	16.2	0.128	23.9	0.357	16.2 (4.66; 37.1)
Intra-operative transfusion volume	0.02	0.900	-0.05	0.928	-0.09	0.897	-58.1 (8.20; 1200)
Sex (Female)	6.23	0.460	-1.99	0.867	-3.55	0.836	1.91 (1.39; 25.2)
Surgical (Total hip replacement)	-54.5	<0.001	-83.5	<0.001	-167	<0.001	83.3 (0.467; 126)
**Intracapsular NOF fractures, patients 70 or above**							
Intercept	101	<0.001	141	<0.001	269	<0.001	
Time to surgery	0.40	0.015	0.38	<0.001	0.10	0.815	4.66 (-1.49; 10.8)
ASA status	9.98	0.101	19.3	0.026	20.8	0.226	36.7 (24.0; 49.3)
CCI	5.76	0.436	16.2	0.128	23.9	0.357	16.6 (8.53; 24.6)
Intra-operative transfusion volume	0.02	0.9	-0.05	0.928	-0.09	0.897	42.7 (-57.6; 143)
Sex (Female)	6.23	0.460	-.199	0.867	03.55	0.836	5.80 (-11.3; 22.9)
Surgical (Total hip replacement)	-54.5	<0.001	-83.5	<0.001	-167	<0.001	-112 (-130; -94.6)
**Extracapsular NOF fractures, patients under 70**							
Intercept	65.4	0.002	37.9	0.290	69.4	0.155	
Time to surgery	0.14	0.784	0.51	0.004	0.49	0.051	12.1 (8.21; 16.1)
ASA status	14.9	0.210	54.1	0.025	67.5	0.051	40.1 (11.8; 68.4)
ASA status (non-linear)	11.4	0.682	-35.5	0.634	93.6	0.347	-
CCI	42.8	0.002	63.3	<0.001	57.8	0.017	63.0 (37.0; 89.0)
Intra-operative transfusion volume	0.05	0.701	0.31	0.135	0.22	<0.001	400 (-115; 915)
Sex (Female)	-4.37	0.578	-10.7	0.516	-11.1	0.742	-4.14 (-36.6; 28.4)
Surgical type (intramedullary nail)	-20.1	0.029	-25.4	0.203	-12.3	0.597	18.8 (-18.1; 55.7)
**Extracapsular NOF fractures, patients 70 or above**							
Intercept	-45.9	0.299	-96.9	0.054	-46.2	0.632	
Time to surgery	0.15	0.129	0.12	0.259	0.19	<0.001	2.48 (-1.82; 6.78)
ASA status	116	<0.001	171	<0.001	205	<0.001	141 (82.9; 200)
ASA status (non-linear)	-126	<0.001	-203	<0.001	-221	0.003	-
CCI	7.45	0.054	24.4	<0.001	39.0	<0.001	24.4 (15.7; 33.0)
Intra-operative transfusion volume	0.05	0.230	-0.01	0.773	0.00	0.979	-7.79 (-60.8; 45.2)
Sex (Female)	15.4	0.175	20.8	0.029	2.97	0.888	-20.8 (-39.5; -2.19)
Surgical type (intramedullary nail)	-8.88	0.393	17.9	0.082	7.28	0.769	-17.9 (-38.1; 2.27)

**The corrected table**:

**Table:** 

Predictor	Coef (tau = 0.25)	P-value (tau = 0.25)	Coef (tau = 0.50)	P-value (tau = 0.5)	Coef (tau = 0.75)	P-value (tau = 0.75)	Effect (95% CI)
**Intracapsular NOF fractures, patients under 70**							
Intercept	101	<0.001	141	<0.001	269	<0.001	
Time to surgery	0.40	0.015	0.38	<0.001	0.10	0.815	8.61 (4.60; 12.6)
ASA status	9.98	0.101	19.3	0.026	20.8	0.226	19.4 (2.42; 36.3)
CCI	5.76	0.436	16.2	0.128	23.9	0.357	16.2 (4.66; 37.1)
Intra-operative transfusion volume	0.02	0.900	-0.05	0.928	-0.09	0.897	-58.1 (8.20; 1200)
Sex (Female)	6.23	0.460	-1.99	0.867	-3.55	0.836	1.91 (1.39; 25.2)
Surgical (Total hip replacement)	-54.5	<0.001	-83.5	<0.001	-167	<0.001	83.3 (0.467; 126)
**Intracapsular NOF fractures, patients 70 or above**							
Intercept	122	<0.001	160	<0.001	187	<0.001	
Time to surgery	0.23	0.073	0.21	0.137	0.41	0.224	4.66 (-1.49; 10.8)
ASA status	20.0	<0.001	36.7	<0.001	63.2	<0.001	36.7 (24.0; 49.3)
CCI	4.38	0.204	16.6	<0.001	37.4	<0.001	16.6 (8.53; 24.6)
Intra-operative transfusion volume	0.02	0.660	0.05	0.404	0.36	0.280	42.7 (-57.6; 143)
Sex (Female)	3.49	0.620	-5.80	0.507	-15.5	0.374	5.80 (-11.3; 22.9)
Surgical (Total hip replacement)	-75.4	<0.001	-112	<0.001	-162	<0.001	-112 (-130; -94.6)
**Extracapsular NOF fractures, patients under 70**							
Intercept	65.4	0.002	37.9	0.290	69.4	0.155	
Time to surgery	0.14	0.784	0.51	0.004	0.49	0.051	12.1 (8.21; 16.1)
ASA status	14.9	0.210	54.1	0.025	67.5	0.051	40.1 (11.8; 68.4)
ASA status (non-linear)	11.4	0.682	-35.5	0.634	93.6	0.347	-
CCI	42.8	0.002	63.3	<0.001	57.8	0.017	63.0 (37.0; 89.0)
Intra-operative transfusion volume	0.05	0.701	0.31	0.135	0.22	<0.001	400 (-115; 915)
Sex (Female)	-4.37	0.578	-10.7	0.516	-11.1	0.742	-4.14 (-36.6; 28.4)
Surgical type (intramedullary nail)	-20.1	0.029	-25.4	0.203	-12.3	0.597	18.8 (-18.1; 55.7)
**Extracapsular NOF fractures, patients 70 or above**							
Intercept	-45.9	0.299	-96.9	0.054	-46.2	0.632	
Time to surgery	0.15	0.129	0.12	0.259	0.19	<0.001	2.48 (-1.82; 6.78)
ASA status	116	<0.001	171	<0.001	205	<0.001	141 (82.9; 200)
ASA status (non-linear)	-126	<0.001	-203	<0.001	-221	0.003	-
CCI	7.45	0.054	24.4	<0.001	39.0	<0.001	24.4 (15.7; 33.0)
Intra-operative transfusion volume	0.05	0.230	-0.01	0.773	0.00	0.979	-7.79 (-60.8; 45.2)
Sex (Female)	15.4	0.175	20.8	0.029	2.97	0.888	-20.8 (-39.5; -2.19)
Surgical type (intramedullary nail)	-8.88	0.393	17.9	0.082	7.28	0.769	-17.9 (-38.1; 2.27)

**Impact on Study Findings**:

This error does not affect the overall results, scientific interpretations, or conclusions of the published study.

We apologise to the readers and the editorial board for any confusion or inconvenience this oversight may have caused.

On behalf of all authors,

Linda Yuhan Tang.

The Original Article has been corrected.

