# Supplementary material for: Time to surgery and its impact on length of stay and mortality for neck of femur fractures at a major trauma centre
Source: Eur J Trauma Emerg Surg. 2026 Aug 24;52(1):261. doi: 10.1007/s00068-026-03313-2 (PMC13503398; doi:10.1007/s00068-026-03313-2)
Supplement: Supplementary file 1 [file 68_2026_3313_MOESM1_ESM.docx]

**Supplementary figure 1:** Prediction plots of ASA status and its effect on the length of stay. Non-linear effects with some extra capsular fractures are shown in the plots

**Supplementary table 1:** General patient characteristic across the subgroups.

Statistical significance is taken at p < 0.05.

| Parameters | Mean (SD) [Intracapsular] | Mean (SD) [Extracapsular] | U -statistic | p-value |
| --- | --- | --- | --- | --- |
| Age | 82.3 (9.27) | 81.8 (12.3) | 2300000 | 0.0276 |
| ASA | 2.82 (0.71) | 2.86 (0.70) | 2210000 | 0.0607 |
| Charlson | 1.46 (1.27) | 1.49 (1.30) | 2360000 | 0.492 |
| Time to surgery | 40.2 (77.5) | 38.7 (75.6) | 2510000 | 0.00351 |
| Length of stay | 336 (272) | 366 (265) | 2120000 | < 0.0001 |
| Intra-operative transfusion volume | 6.60 (56.8) | 28.5 (120) | 2280000 | < 0.0001 |

**Supplementary table 2**

(a) Mean and standard deviation of age on admission, ASA status, CCI, time to surgery and transfusion volume in intracapsular and extracapsular fractures, stratified by mortality.

| Comparator | Mean (SD) [Alive] | Mean (SD) [Dead] | U-statistic | p-value |
| --- | --- | --- | --- | --- |
| Age on admission (Intracapsular) | 82.1 (9.30) | 86.4 (7.31) | 89000 | < 0.0001 |
| Age on admission (Extracapsular) | 81.5 (12.5) | 86.6 (7.31) | 74800 | < 0.0001 |
| ASA status (Intracapsular) | 2.80 (0.701) | 3.41 (0.554) | 62600 | < 0.0001 |
| ASA status (Extracapsular) | 2.83 (0.70) | 3.35 (0.534) | 58500 | < 0.0001 |
| CCI (Intracapsular) | 1.40 (1.24) | 2.80 (1.21) | 51100 | < 0.0001 |
| CCI (Extracapsular) | 1.43 (1.27) | 2.48 (1.28) | 55100 | < 0.0001 |
| Time to surgery (Intracapsular) | 38.50 (73.7) | 80.9 (135) | 89500 | < 0.0001 |
| Time to surgery (Extracapsular) | 38.3 (76.7) | 47.7 (55.1) | 80800 | 0.00123 |
| Intra-operative transfusion volume (Intracapsular) | 6.18 (56.3) | 16.7 (67.2) | 116000 | 0.000440 |
| Intra-operative transfusion volume (Extracapsular) | 27.2 (118) | 49.7 (145) | 92400 | 0.00618 |

(b) Number of patients for each surgical type stratified by mortality and fracture type.

| Comparator | Number (Alive) | Number (Dead) |
| --- | --- | --- |
| **Intracapsular** | | |
| Hemiarthroplasty | 2123 | 99 |
| Total hip replacement | 283 | 2 |
| **Extracapsular** | | |
| Screw | 1479 | 91 |
| Intramedullary nail | 319 | 19 |

**Supplementary table 3:** Chi-Square test comparing admission death between the under 36h and above 36h time to surgery groups. Statistical significance is taken as p < 0.05.

| Comparator | Proportions (≤36h) | Proportions (>36h) | Chi-Square value | p-value |
| --- | --- | --- | --- | --- |
| Death (Intracapsular) | Yes: 42, No: 1568 | Yes: 59, No: 838 | 22.5 | < 0.0001 |
| Death (Extracapsular) | Yes: 62, No: 1222 | Yes: 48, No: 576 | 5.08 | 0.02 |

**Supplementary table 4:**

(a) Mann-Whitney U test comparing the different parameters for the below 36 hours and above 36 hours groups. Statistical significance is taken at p < 0.05

| Comparator | Mean (SD) [≤ 36 hours] | Mean (SD) [> 36h] | U-statistic | p-value |
| --- | --- | --- | --- | --- |
| Age on admission (Intracapsular) | 82.5 (9.15) | 81.8 (9.46) | 688000 | 0.0505 |
| Age on admission (Extracapsular) | 82.0 (12.6) | 81.4 (11.8) | 383000 | 0.110 |
| ASA status (Intracapsular) | 2.78 (0.679) | 2.90 (0.745) | 754000 | 0.000340 |
| ASA status (Extracapsular) | 2.80 (0.69) | 2.97 (0.698) | 427000 | < 0.0001 |
| CCI (Intracapsular) | 1.40 (1.22) | 1.57 (1.35) | 766000 | 0.00893 |
| CCI (Extracapsular) | 1.41 (1.28) | 1.66 (1.31) | 446000 | < 0.0001 |
| Time of stay (Intracapsular) | 334 (267) | 341 (282) | 728000 | 0.742 |
| Time of stay (Extracapsular) | 361 (262) | 374 (271) | 415000 | 0.205 |
| Intra-operative transfusion volume (Intracapsular) | 5.82 (52.4) | 8.01 (64.1) | 727000 | 0.223 |
| Intra-operative transfusion volume (Extracapsular) | 29.9 (121) | 25.5 (117) | 396000 | 0.326 |

(b) Number of patients for each surgical type stratified by time to surgery

| Comparator | Number (≤ 36h) | Number (> 36h) |
| --- | --- | --- |
| **Intracapsular** | | |
| Hemiarthroplasty | 1451 | 771 |
| Total hip replacement | 159 | 126 |
| **Extracapsular** | | |
| Screw | 1075 | 495 |
| Intramedullary nail | 209 | 129 |

**Supplementary table 5:**

(a) Mann-Whitney U test comparing the different parameters for the under 70 and 70 or above age groups. Statistical significance is taken at p < 0.05

| Comparator | Mean (SD) [Under 70] | Mean (SD) [70 or above] | U-statistic | p-value |
| --- | --- | --- | --- | --- |
| ASA status (Intracapsular) | 2.3 (0.843) | 2.88 (0.67) | 163000 | < 0.0001 |
| ASA status (Extracapsular) | 2.25 (0.84) | 2.95 (0.628) | 106000 | < 0.0001 |
| CCI (Intracapsular) | 0.822 (1.03) | 1.53 (1.28) | 182000 | < 0.0001 |
| CCI (Extracapsular) | 0.755 (1.04) | 1.60 (1.29) | 122000 | < 0.0001 |
| Time to surgery (Intracapsular) | 38.9 (31.5) | 40.3 (80.9) | 291000 | 0.111 |
| Time to surgery (Extracapsular) | 44.0 (88.0) | 37.9 (73.6) | 206000 | 0.765 |
| Time of stay (Intracapsular) | 214 (204) | 349 (276) | 151000 | < 0.0001 |
| Time of stay (Extracapsular) | 250 (247) | 383 (264) | 116000 | < 0.0001 |
| Intra-operative transfusion volume (Intracapsular) | 10.8 (83.1) | 6.16 (53.3) | 277000 | 0.251 |
| Intra-operative transfusion volume (Extracapsular) | 21.6 (119) | 29.5 (120) | 198000 | 0.121 |

(b) Number of patients for each surgical type stratified by age

| Comparator | Number (Under 70) | Number (Over 70) |
| --- | --- | --- |
| Intracapsular | | |
| Hemiarthroplasty | 114 | 2108 |
| Total hip replacement | 128 | 157 |
| Extracapsular | | |
| Screw | 181 | 1389 |
| Intramedullary nail | 64 | 274 |
